# Toward a Smartphone-Based and Conversational Agent–Delivered Just-in-Time Adaptive Holistic Lifestyle Intervention for Older Adults Affected by Cognitive Decline: Two-Week Proof-of-Concept Study

**DOI:** 10.2196/66885

**Published:** 2025-07-28

**Authors:** Esther Brill, Rasita Vinay, Marcia Nißen, Priyam Joshi, Stefan Klöppel, Tobias Kowatsch

**Affiliations:** 1 University Hospital of Old Age Psychiatry and Psychotherapy, University of Bern, Bern, Switzerland; 2 Institute of Biomedical Ethics and History of Medicine, University of Zurich, Zurich, Switzerland; 3 School of Medicine, University of St. Gallen, St. Gallen, Switzerland; 4 Centre for Digital Health Interventions, Department of Management, Technology, and Economics, ETH Zurich, Weinbergstrasse 56/58, Zurich, 8006, Switzerland, +41 446329652; 5 Institute for Implementation Science in Health Care, University of Zurich, Zurich, Switzerland; 6 Department of Psychology, Georgia State University, Atlanta, GA, United States

**Keywords:** conversational agents, older adults, just-in-time adaptive intervention, health intervention, smartphone-based, mHealth, mobile health, digital health, smartphone, digital, technology, single-arm feasibility study, feasibility, dementia, global health, cognitive impairment, awareness, dementia prevention, digital health interventions, older person, aging, mobile phone

## Abstract

**Background:**

Dementia is projected to impact 152 million people by 2050, making it one of the most pressing global health challenges. The neurodegenerative process initiates well before clinical symptoms manifest, advancing from subjective cognitive decline (SCD) to mild cognitive impairment (MCI) and ultimately to dementia. Despite the growing prevalence, awareness of dementia prevention is limited, and many individuals express a desire to cease living upon diagnosis. Lifestyle interventions can mitigate cognitive decline, but there is a need for effective, scalable approaches to deliver these interventions to older adults. Digital health interventions, such as app-based just-in-time adaptive interventions, offer a promising solution, but their application in cognitively impaired older populations remains underexplored.

**Objective:**

This formative study evaluated the plausibility, acceptability, and adherence to a smartphone-based just-in-time adaptive digital lifestyle intervention delivered by a rule-based conversational agent (CA) among older adults with SCD or MCI. The primary focus was on adherence to the CA-initiated conversational turns (measured objectively via interaction logs), and secondary objectives included perceptions of technology acceptance, working alliance with the CA, self-reported adherence to the suggested health-promoting activity, and feedback for future improvements (through a questionnaire and short interview).

**Methods:**

This monocentric study investigated 15 participants (mean age 70.3, SD 5.01; 10 female and 5 male participants) with SCD (n=12) or MCI (n=3). Participants used the study app that delivered daily health-promoting activities through a CA over 2 weeks. Participants received notifications to engage in 7 health-related activities, and adherence to the activities was self-reported. Post intervention, participants rated their experience with the app and assessed their working alliance with the CA through the 6-item session alliance inventory. Data on smartphone use, demographic information, and cognitive performance (via Montreal Cognitive Assessment) were collected during a preintervention visit.

**Results:**

Participants rated the study app positively, especially regarding ease of use and a subset of the working alliance. Adherence to the CA-initiated conversational turn was measured at an average of 81% across 14 days. In total, 27% (mean 4.07, SD 2.27) of participants indicated being vulnerable, and 100% then responded with their state of receptivity, of which 83% (mean 3.14, SD 1.61) were receptive to completing the activity, and 69% (mean 2.86, SD 1.70) self-reported adherence to the activity. There was no significant decline in adherence across the study period. Qualitative results support these findings and present two emerging themes: app enjoyment and enhancing engagement.

**Conclusions:**

This study demonstrates that smartphone-based just-in-time adaptive interventions are feasible and generally well-accepted by older adults with SCD or MCI. However, the findings underscore the need for robust technological infrastructure and potential personal assistance to optimize adherence. Future interventions could benefit from integrating wearables to improve real-time engagement and accurately monitor adherence, ultimately supporting healthy aging and cognitive health in older populations.

## Introduction

Dementia is expected to become one of the most critical global health challenges, with an estimated prevalence of 152 million patients worldwide by 2050 [1]. The neurodegenerative cascade in dementia commences well before clinically significant symptoms appear and can be conceptualized as a continuum extending from subjective cognitive decline (SCD) in the preclinical phase to mild cognitive impairment (MCI) to more advanced stages of the disease [2]. Recent international data suggest that approximately one in four individuals aged 60 years and older experience SCD [3], representing a significant risk factor with a subsequent conversion rate of 27% from SCD to MCI and a 14% progression from SCD to dementia within a decade [4,5]. Crucially, in Switzerland, 25% of individuals surveyed by the Swiss “Demenzbarometer” expressed a preference to cease living upon a dementia diagnosis, reflecting profound personal and societal implications [6].

Despite growing evidence from high-quality randomized controlled trials that indicates that lifestyle interventions can improve or maintain quality of life (QoL), slow cognitive decline, and even mitigate epigenetic age acceleration [7-9], awareness of dementia prevention remains limited: one-third of individuals living in Switzerland are unaware that lifestyle choices can impact dementia risk [6]—a gap underscoring the need for accessible, scalable approaches that promote health literacy and support everyday health behavior change.

Indeed, in recent years, a growing number of mobile and digital health interventions [10,11] have been developed to address modifiable risk factors associated with cognitive decline. Notable examples include the Maintain Your Brain platform [12-14] or the GrayMatters app [15-17], which combines psychoeducational content with self-tracking options across multiple domains such as nutrition, cognition, physical activity, and sleep. While these interventions show promising results (eg, with regard to cognitive decline, diet quality, and physical activity), they are often static, requiring high user initiative and offering limited real-time personalization or behavioral guidance adapted to an individual’s changing needs or contexts [18].

Smartphone-based, just-in-time adaptive interventions (JITAIs) offer a promising alternative for delivering personalized behavioral support when individuals need it [19]. JITAIs are “interventions that adapt over time to an individual’s changing status and circumstances with the goal to address the individual’s need for support, whenever this need arises” [20]. They are, by definition, designed to provide tailored support to individuals during states of vulnerability or opportunity (eg, moments of adverse health behavior), receptivity (eg, when they can receive and process the support provided), and adherence (eg, using or adopting the support) [19,21]. They are also considered underdeveloped and underresearched, with a recent review identifying them as “a significant missed opportunity” for tailoring digital interventions [18,22].

While early research on JITAIs has demonstrated some efficacy in targeting lifestyle behaviors [23,24], they have so far primarily been designed and tested in young, healthy, and cognitively intact populations [25,26] in diverse settings or among different demographic groups [27]. Yet, their implementation in older populations affected by cognitive decline, especially given the added barriers of reduced digital literacy and potential accessibility constraints [28,29], presents unique challenges. Individuals with SCD or MCI may experience difficulties with abstract app navigation or complex content, which can compromise engagement [30].

In this context, relational agents—software that is designed to emulate conversations through voice or SMS text messages, also known as chatbots or digital coaches [31]—offer a compelling interface for delivering JITAIs. Relational agents have been shown to foster therapeutic rapport with users [32-34], support long-term engagement [35], and have been deployed across various clinical [35-37] and nonclinical populations [38-40], including (cognitively healthy) older adults [41-43]. However, most relational agent studies have been domain-specific (eg, targeting physical activity [41] or adherence to home blood pressure monitoring [44]), and have not been integrated into a broader JITAI framework to deliver holistic, multidomain sets of lifestyle interventions tailored to older adults with cognitive decline.

Taken together, the potential for combining (1) JITAI logic and (2) relational agents for the delivery of a (3) holistic, multidomain lifestyle intervention in (4) particularly underserved populations, such as older adults with SCD or MCI—remains completely underexplored.

To address this gap, this study explores the plausibility of a smartphone-based and relational agent–delivered just-in-time adaptive holistic lifestyle intervention in older adults with SCD or MCI, guided by the following research questions (RQs): (RQ1) To what extent do older adults with SCD or MCI adhere to a smartphone-based, CA-delivered JITAI over a 2-week period? (RQ2) How do participants perceive the usability and acceptability of the intervention?

By answering these questions, this study will contribute to the emerging research space at the intersection of digital lifestyle interventions, JITAIs, and relational agents—and thus to the integration of critical but often siloed research areas—by providing insights into how such tools can be adapted for cognitively vulnerable, aging populations.

## Methods

### Study Design

This study was designed as a 2-week, single-arm proof-of-concept study to evaluate the acceptability, adherence, and technical implementation of a smartphone-based, holistic just-in-time adaptive digital lifestyle intervention delivered by a rule-based CA. The interventions focused on promoting daily health behaviors across 7 domains relevant to cognitive and overall health, including physical activity, nutrition, sleep, cognitive exercises, hydration, and social engagement. The target population consisted of older adults with either SCD or MCI.

As a proof-of-concept study, the primary objectives were to assess plausibility, acceptability, and adherence patterns to the CA-initiated dialog sequences, and participants’ subjective evaluation of the intervention’s usability and acceptability. Secondary objectives included exploring the perceived working alliance with the CA and collecting qualitative feedback to inform future iterations of the intervention.

### Intervention Design

We adopted the JITAI framework to design a low-burden, chatbot-delivered digital lifestyle intervention tailored to the behavioral needs and capacities of older adults with SCD or MCI—individuals in the early stages of cognitive decline who generally retain the ability to provide reliable, momentary self-reports when prompted with low-burden, structured questions and limited recall windows [45].

Following the conceptual framework by Nahum-Shani et al [20], which defines key elements such as decision points, tailoring variables, intervention options, and decision rules, and proximal or distal outcomes (Table 1), the intervention was designed to assess a participant’s momentary states and only deliver a lifestyle suggestion when two criteria were met: (1) the participant had not yet engaged in the suggested lifestyle behavior (ie, determining the state of vulnerability or opportunity), and (2) the participant indicated availability and willingness to engage in the suggested lifestyle behavior (ie, state of receptivity).

**Table 1.** Key terms of a JITAI^a^ and its operationalization^b^ to the Elsa/Erik^c^ study among older adults with SCD^d^ or MCI^e^.

Key term	Operationalization
Distal outcome	For example, quality of life and cognitive health.
Proximal outcomes	Implementation (yes/no) of a specific daily lifestyle behavior, such as a walk in the park or an activity nutrient-rich diet.
State of vulnerability or opportunity	An individual is classified as vulnerable by the CA^f^ Elsa/Erik (yes/no), if a specific health-promoting lifestyle behavior (see Table 2 for all behaviors) has not been implemented until the early afternoon (see Decision points below);
State of receptivity	An individual is classified as receptive and open to implementing a health-promoting lifestyle suggestion (yes/no) by the CA Elsa/Erik, if she or he has time in the afternoon or early evening to implement such a behavior.
Decision points	Every day in the early afternoon, either 1 PM, 2 PM, or 3 PM; the specific time when Elsa/Erik should ask the participant about the state of vulnerability and receptivity was defined by the study participant during the first conversational turns with Elsa/Erik.
Intervention options	Low-burden, easy-to-implement lifestyle interventions focusing on diet and nutrition (eg, fruits and vegetables), physical activity (eg, a walk in the park), sleep (eg, 8 h of uninterrupted sleep), stress management, etc (see Table 2 for a full list of the options) recommended by Elsa/Erik if someone was classified as vulnerable and receptive.
Tailoring variables	Participants’ response (yes/no) to her or his state of vulnerability and receptivity across a predefined lifestyle behavior (eg, if the target person has not yet implemented a health-promoting behavior such as physical activity, a variety of physical activity options would have been suggested by Elsa/Erik).
Decision rules	If someone is vulnerable (see tailoring variables above) and if that person also indicates that she or he is receptive to implementing a suggested behavior, then the intervention option that matches the vulnerable state is delivered by Elsa/Erik; also refer to the visualization of the JITAI logic in Figure 1.

a JITAI: just-in-time adaptive intervention.

b Table outline adapted from Nahum-Shani et al [19].

c Elsa/Erik was a 2-week proof-of-concept study, and we were primarily interested in the response rates to the conversational agent and the adherence to the recommended lifestyle behaviors as proximal outcomes. We did not expect effects on potential distal outcomes.

d SCD: subjective cognitive decline.

e MCI: mild cognitive impairment.

f CA: conversational agent.

**Table 2.** An overview of the 7 health-promoting activities prompted through the CAs^a^, Elsa/Erik, during the 2-week single-arm proof-of-concept study among older adults with SCD^b^ or MCI^c^.

Aspect of health	Example
Days 1 and 8: Exercise	Moderate exercise such as 30 minutes of walking, swimming, and cycling
Days 2 and 9: Balanced diet	Eating 4 different types of fruit and vegetables throughout the day
Days 3 and 10: Sleep	Sleep for 8 hours the past night, if not, nap for 25 minutes
Days 4 and 11: Cognitive exercise	Crossword puzzle, sudoku, scrabble
Days 5 and 12: Movement	Bike ride, work in the garden, dancing, yoga
Days 6 and 13: Social engagement	Meet or call a close friend or relative
Days 7 and 14: Hydration	Drinking 5 glasses (0.2 L) of water

a CA: conversational agent.

b SCD: subjective cognitive decline.

c MCI: mild cognitive impairment.

**Figure 1.**
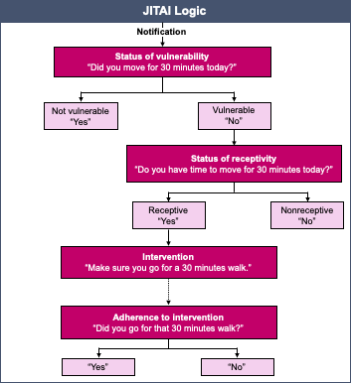
A decision tree indicating the JITAI logic for this 2-week single-arm proof-of-concept study among older adults with SCD or MCI, delivered by a rule-based CA through a smartphone app. CA: conversational agent; JITAI: just-in-time adaptive intervention; MCI: mild cognitive impairment; SCD: subjective cognitive decline.

Each day’s JITAI was structured around a decision point, occurring at a time predefined by each participant (1 PM, 2 PM, or 3 PM). At this time, the CA first assessed the participant’s state of vulnerability—a key tailoring variable—by asking whether they had already completed the targeted activity (eg, physical activity, hydration, or social contact).

If vulnerability (=an opportunity) was detected, because the response was “no,” a second tailoring variable—the participant’s state of receptivity was assessed to determine a second decision point. Therefore, the CA asked whether the individuals felt able and willing to complete the suggested activity later that day. Only when both vulnerability and receptivity were affirmed, the intervention option was triggered: a low-burden, tailored suggestion encouraging engagement in a target behavior (eg, “Why don’t you try and go for a 30-min walk. I’m sure you’ll feel great after”).

A third decision point occurred in the evening, where the CA inquired whether the participant had completed the suggested behavior. This response was used to capture the proximal outcome of adherence—that is, whether the support had led to real-world action that day.

Given the short duration and early-phase nature of this proof-of-concept, no distal outcome (eg, change in the QoL or long-term cognitive functioning) was assessed at this stage.

The complete mapping of each JITAI component to its operationalization in this study is also summarized in Table 1 and visualized in Figure 1. This table further clarifies how each core construct—decision points, tailoring variables, intervention options, decision rules, and outcomes—was instantiated within the JITAI logic of the Elsa/Erik study.

### Technical Implementation

**Figure 2.**
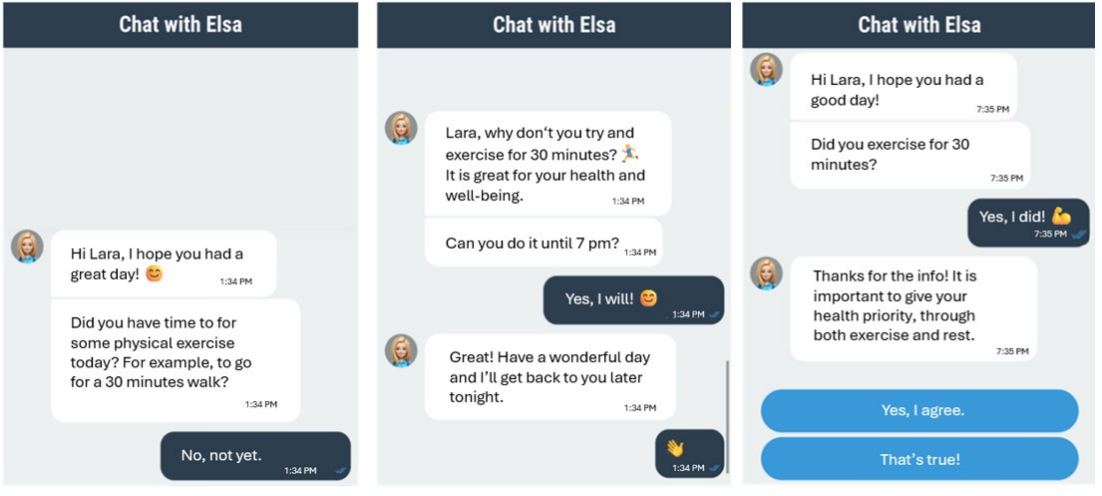
The three conversational turns with the CA Elsa providing the JITAI in the 2-week single-arm proof-of-concept study among older adults with SCD or MCI. Translated from German. From left to right; first chat panel: determining the state of vulnerability (ie, “Have you completed an activity today?”); second chat panel: determining the state of receptivity (ie, if no: “Would you be able to complete the activity later?”); and third chat panel: determining adherence to the CA-suggested health-promoting activity (ie, “Did you complete this activity today?”). CA: conversational agent; JITAI: just-in-time adaptive intervention; MCI: mild cognitive impairment; SCD: subjective cognitive decline.

The smartphone-based JITAI was developed with the latest version of the MobileCoach software platform (April 2024) [46,47], which has already been used for various health interventions [31,37,39,40,48-51]. MobileCoach offers data collection capabilities (eg, via self-reports and interaction logs) and the delivery of JITAIs with the help of a rule-based CA. The user interface follows the principles of messaging apps, such as Apple’s iMessage or WhatsApp. However, answer options are predominantly predefined with a few exceptions (eg, when the CA asks for the nickname of the study participant). MobileCoach was hosted by Pathmate Technologies. Participants could choose from two CAs, Elsa or Erik, to be their digital coach over the 2-week study period. Figure 2 demonstrates the three conversational turns identifying the state of vulnerability, state of receptivity, and adherence to the CA-suggested health-promoting activity.

### Recruitment

Participants were recruited in the Bern Memory Clinic and local cognitive training groups [52] in July 2024. First, a telephone screening for a first check of inclusion and exclusion criteria and a detailed explanation of the study was performed. An investigator explained to each participant the nature of the study, its purpose, the procedures involved, the expected duration, the potential risks and benefits, and any discomfort it may entail. As inclusion criteria, participants were required to be aged between 60 and 85 years, exhibit cognitive decline classified as SCD or MCI, possess a smartphone (either iOS or Android operated), demonstrate fluency in German, and have the capacity to provide written informed consent. Participants were excluded if they had severe psychiatric disorders or a history of substance abuse. Cognitive performance was assessed using the Montreal Cognitive Assessment (MoCA) score, with >25 indicating SCD, and scores equal to or below 25 indicating MCI [53,54] by expert investigators in a standardized clinical setting. Detailed study information and consent forms were sent to potential participants several days prior to the on-site visit. Participants gave written informed consent and were informed of their right to withdraw from the study at any time. Participation was voluntary, and no incentive for participation was provided.

### Procedure

During the on-site preintervention visit, demographic data, general cognitive performance (assessed using the MoCA Score), and technology readiness were evaluated. Questions on technological readiness included the duration, modality, and frequency of smartphone use (Multimedia Appendix 1).

Participants then downloaded the study app, MobileCoach, and received 2 weeks of JITAI promoting healthy lifestyle behaviors through their CA. The CA started a maximum of two daily dialogs with the participant, one in the afternoon (to assess the state of vulnerability and receptivity) and one in the evening (to assess whether the health-promoting activity was implemented) if the participant indicated that they were vulnerable in the afternoon. Participants preselected their preferred times for these dialogs with the CA: 1 PM, 2 PM, or 3 PM for the afternoon dialog and 7 PM, 8 PM, or 9 PM for the evening dialog. The notifications prompted health-promoting activities and assessed the participant’s vulnerability and receptivity to these activities. In total, the CA suggested health-promoting activities from 7 aspects of health, twice each during the 2-week study period (Table 2). The health-promoting activities were decided among three authors (EB, RV, and PJ), based on evidence-based domains affecting positive health outcomes in the literature [55].

After the 2-week study period, a follow-up telephone call was conducted to confirm study completion and gather feedback on app use. Participants were sent (digitally or by post) a postintervention questionnaire (Multimedia Appendix 2).

### Data Collection

The primary end point is adherence to the CA-initiated conversational turns, measured objectively via app interaction logs. Data from the app were collected using the MobileCoach software platform in anonymized form using participant IDs. The platform allowed the data to be exported and further used for analyzing the measured metrics reported in the results section. States of vulnerability, receptivity, and adherence to both the CA-initiated conversational turns and CA-suggested health-promoting activities, as well as the daily response rates to vulnerability questions, were measured.

Secondary end points include perceptions of technology acceptance, working alliance with the CA, self-reported adherence to the CA-suggested health-promoting activity, and suggestions for improving the JITAI (conducted through a questionnaire and short telephone interview). As a clarification, adherence to the CA-initiated conversational turns refers to whether the participants responded to the first notification assessing their state of vulnerability. Adherence to the CA-suggested health-promoting activity refers to whether the participant was able to complete the suggested activity.

A postintervention questionnaire was used to assess technology acceptance constructs, based on prior work on CA [31]. Constructs measured in this study included perceived ease of use, enjoyment, usefulness (as a reminder of the health-promoting behavior and as a motivator to complete the activity), control over the CA, and also the intention to interact again with the CA [56-58]. Working alliance, an important relationship quality, has previously been shown to relate strongly to treatment outcomes [59-62] and was measured with the 6-item session alliance inventory [63]. A 7-point Likert scale was used for each item and anchored from 1=strongly disagree to 7=strongly agree.

During the follow-up telephone call, a short semistructured interview was conducted to ask participants: (1) What did you particularly like about the interaction with Elsa/Erik? (2) What needs to be improved about the interaction with Elsa/Erik? (3) Do you have any further comments or suggestions for improvement? The postintervention questionnaire also included these three open-ended questions, which were then updated and added to their interview responses. Additionally, participants were also provided with space to provide open feedback and details regarding their smartphone and mobile operating system.

### Data Analysis

Based on the mobile app data collected by MobileCoach, metrics for vulnerability, receptivity, and both adherences (ie, CA-initiated conversational turns and CA-suggested health-promoting activity) could be analyzed. Adherence to the CA-initiated conversational turns was calculated from the response to notifications on the status of vulnerability—the first afternoon message. Participants were categorized as vulnerable (who had not yet completed the activity), not vulnerable (who had already completed the activity), or nonresponsive. Receptivity was assessed only among vulnerable participants, who were further categorized based on their intent to complete the activity. Participants were either classified as receptive (intended to complete the activity), not receptive (did not intend to complete the activity), or nonresponsive. Proportions were calculated by dividing the number of participants in each category by the total number of vulnerable participants. Adherence to the CA-suggested health-promoting activity was measured by tracking whether participants who were vulnerable in the afternoon completed the activity by the evening notification time. Among these participants, those who completed the activity were considered to have adhered, while those who did not were categorized as not adhered. The proportion of adherence was calculated by dividing the number of participants who completed the activity by the total number of vulnerable participants who responded to the evening message.

For the self-report items of the postintervention questionnaires, we conducted descriptive statistics to report mean and SD calculations. Shapiro-Wilk tests indicated that the self-reported data were not normally distributed, and therefore, Wilcoxon signed rank tests were conducted to assess whether the median of the evaluations significantly differed from the test value of 4, representing a neutral response on the 7-point Likert scale. An overall score for the 6-item session alliance inventory was computed by averaging the six individual items, as Cronbach α of 0.90 demonstrated high internal consistency [9].

Inductive thematic analysis was conducted with the interview responses using MAXQDA (VERBI) [64]. Codes were determined from the raw questionnaire and interview data (translated into English) by authors EB and RV and further classified into overarching themes based on their trends. Two rounds of thematic analysis were conducted, and the results are presented as code and thematic maps, also using MAXQDA.

### Ethical Considerations

The study was reviewed by the local Ethics Committee in Bern (identifier 2024‐01029) and categorized as not falling under the Human Research Act [65]. Written informed consent was obtained from all participants in the study language, German. Participants were sent a study information sheet that included the inclusion and exclusion criteria, detailed information about the study, and their rights as participants, as well as a consent form for participation in the study and a consent form for further use of data. All data collected in this study were anonymized, and no identification of individual participants in the data, study, or supplementary files is possible. Collection, processing, and storage procedures for this study strictly adhered to Swiss legal and ethical frameworks, including the Swiss Federal Act on Research involving Human Beings (Human Research Act) and the new Federal Act on Data Protection. Participants were not provided compensation for the study.

## Results

### Characteristics of the Study Population and Technology Readiness

Overall, 17 participants were recruited for the study. One participant was excluded due to outdated hardware, and another participant due to technical issues (notifications could not be delivered), resulting in a total analyzable sample of 15 participants. All participants were assigned to the CA-delivered app intervention in this single-arm study, and reporting followed the CONSORT (Consolidated Standards of Reporting Trials) checklist (Checklist 1). Participant sample characteristics indicated 67% (n=10) identified as female, and 33% (n=5) as male, where the mean age was 70.3 (SD 5.01) years, and the mean MoCA Score was 27.73 (SD 2.01). Based on the suggested cut-off [53,54], 12 participants were classified as having SCD and 3 as MCI. There was no significant difference between SCD and MCI participants in any of the technology readiness outcomes described in Table 3.

**Table 3.** Data collected from the 2-week, single-arm proof-of-concept study among older adults with SCD^a^ or MCI^b^, showing information on smartphone use and frequency, including the technology readiness of participants.

	Value	
Smartphone and mobile operating system, n (%)		
Android smartphone	8 (53)	
Apple iPhone	7 (47)	
Frequency of carrying the smartphone, n (%)		
Always (including at home)	5 (33)	
When leaving the house	8 (53)	
Only when needed	2 (13)	
Using the smartphone (on a 5-point Likert scale, where 1 is “never” and 6 is “multiple times per day”), mean (SD)		
Daily use for		
Chatting	4.26 (0.88)	
Researching information on the internet	4 (1.3)	
Checking the weather	3.66 (1.11)	
Multiple times per week for		
Phone calls	3.46 (1.06)	
Reading emails	3.46 (1.68)	
Taking photos	3.13 (0.99)	
Consulting their calendar	2.87 (1.77)	
Planning routes	2.8 (1.42)	
Writing emails	2.67 (1.59)	
Once per week for		
Tracking health-related data	2.46 (1.72)	
Listening to music	2.2 (0.94)	
Playing games	1.8 (1.2)	

a SCD: subjective cognitive decline.

b MCI: mild cognitive impairment.

### Response Rates to the JITAI

**Figure 3.**
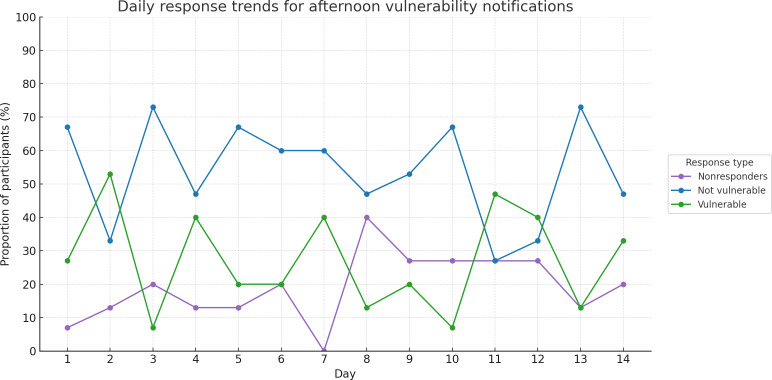
Daily response rates for afternoon vulnerability notifications to determine whether the participant has already completed the suggested activity or not, or if the participant does not respond. Graphed over the 2-week single-arm proof-of-concept study among older adults with subjective cognitive decline or mild cognitive impairment.

On average, 81% (mean 12.14) of participants responded to the afternoon notification each day over the 14-day study period (SD 1.51 responses per day to the state of vulnerability dialogue), demonstrating adherence to the CA-initiated conversational turns. Conversely, 19% (mean 2.86, SD 1.51) of participants, on average, did not respond each day. A detailed breakdown of the daily response rates according to the response type is presented in Figure 3.

For each aspect of health, participant engagement was analyzed across three key stages: determining vulnerability, identifying receptivity, and measuring adherence to the CA-suggested health-promoting activity. Table 4 presents these metrics as percentages, with results grouped by specific activities. On average, 27% (mean 4.07, SD 2.27) of participants reported being vulnerable, and of these, 100% responded to the state of receptivity notification. An average of 83% (mean 3.14, SD 1.61) of participants were receptive to completing the health-promoting activity, with 69% (mean 2.86, SD 1.70) of participants self-reporting adherence to the activity. An average of 95% (mean 3.93, SD 2.27) of participants responded to the adherence evening notification.

**Table 4.** Data collected from the 2-week, single-arm proof-of-concept study among older adults with SCD^a^ or MCI^b^, showing results of participants’ vulnerability, receptivity, and adherence to the CA^c^-suggested health-promoting activity, as proportion (%) and absolute values (n).

	State of vulnerability			State of receptivity			Adherence		
Health-promoting activity	No response^d^	Not vulnerable^e^	Was vulnerable^f^	No response^g^	Was not receptive^h^	Was receptive^i^	No response^j^	Did not adhere^k^	Did adhere^l^
Exercise (%)	23.30	56.70	20	0	0	100	0	37.50	62.50
Day 1 (n)	1	10	4	0	0	4	0	1	3
Day 8 (n)	6	7	2	0	0	2	0	1	1
Balanced diet (%)	20	43.30	36.70	0	25	75	0	25	75
Day 2 (n)	2	5	8	0	4	4	0	4	4
Day 9 (n)	4	8	3	0	0	3	0	0	3
Sleep (%)	23.30	70	6.70	0	0	100	0	50	50
Day 3 (n)	3	11	1	0	0	1	0	1	0
Day 10 (n)	4	10	1	0	0	1	0	0	1
Cognitive exercise (%)	20	36.70	43.30	0	29.80	70.20	7.10	38.10	54.80
Day 4 (n)	2	7	6	0	1	5	0	2	4
Day 11 (n)	4	4	7	0	3	4	1	3	3
Movement (%)	20	50	30	0	25	75	0	16.70	83.30
Day 5 (n)	2	10	3	0	0	3	0	0	3
Day 12 (n)	4	5	6	0	3	3	0	2	4
Social engagement (%)	16.70	66.70	16.70	0	41.70	58.30	25	16.70	58.30
Day 6 (n)	3	9	3	0	1	2	0	1	2
Day 13 (n)	2	11	2	0	1	1	1	0	1
Hydration (%)	10	53.30	36.70	0	0	100	0	0	100
Day 7 (n)	0	9	6	0	0	6	0	0	6
Day 14 (n)	3	7	5	0	0	5	0	0	5

a SCD: subjective cognitive decline.

b MCI: mild cognitive impairment.

c CA: conversational agent.

d This is the proportion of participants who did not respond to the vulnerability message for each intervention type, relative to the total participants for that interventio.n

e This is the proportion of participants who indicated they were “not vulnerable” and had already completed the activity, as a percentage of the total participants for the intervention type.

f This proportion represents participants who indicated they were “vulnerable” for the activity type, again relative to the total participants for that intervention.

g This shows the percentage of participants who did not respond to the receptivity message after indicating vulnerability.

h The proportion of participants who were “not receptive” to completing the activity, among those who were “vulnerable.”

i This is the percentage of vulnerable participants who were “receptive” to completing the activity.

j This represents the percentage of participants who did not respond to the evening check-in for adherence, calculated only for participants who were marked as “vulnerable.”

k The proportion of participants who did “not adhere” to the activity, again calculated only for those marked as “vulnerable.”

l This is the percentage of participants who “adhered” to the activity, calculated based on those who were “vulnerable.”

However, engagement patterns varied significantly across health domains. For example, the cognitive exercise domain showed the highest combined engagement: 43% (n=6.5) of participants reported being vulnerable, 70% (n=4.5) were receptive, and 55% (n=3.5) adhered to the suggested activity. In contrast, sleep was the domain with the lowest proportion of vulnerability: only 1 (7% of all participants) person indicated being vulnerable at all who was receptive on both days and consistently reported that they had already engaged in the recommended sleep behavior by the time the just-in-time prompt was delivered. This suggests that many participants already had stable evening routines or did not perceive a need for behavioral adjustment in this area.

### Technology Acceptance and Working Alliance Assessment

Participants were asked to rate their experience interacting with the CAs Elsa/Erik on a 7-point scale (where 1=strongly disagree and 7 =strongly agree). Further, participants rated their perceived relationship with Elsa/Erik on a 7-point scale (where 1=never and 7=always). The results are displayed in Table 5.

**Table 5.** Data collected from the 2-week, single-arm proof-of-concept study among older adults with SCD^a^ or MCI^b^, showing the postinteraction assessment of 15 participants on PEU^c^, PU^d^, PEN^e^, PC^f^, ITI^g^, and PSA^h^.

#Item		Mean (SD)^i^		V^j^
PEU: I thought the response from Erik/Elsa was easy to understand.		6.60 (0.83)		105.0^k^
PU1: I found it useful to work on the intervention with Erik/Elsa.		5.13 (1.77)		76.0^l^
PU2: I found Erik/Elsa motivated me to perform the exercises.		4.67 (2.02)		55.0
PEN: I had fun using Erik/Elsa.		5.20 (2.08)		85.5^l^
PC: I was able to control the interaction with Erik/Elsa.		5.33 (2.38)		90.0
ITI: I would continue interacting with Erik/Elsa.		4.40 (2.53)		61.0
PSA				
I felt that Erik/Elsa and I respected each other.		6.20 (1.42)		89.5^m^
I felt that Erik/Elsa appreciated me.		6.07 (1.44)		115.0^m^
I felt that Erik/Elsa cared about me even when I did things it did not approve of.		5.67 (1.50)		98.0^m^
I felt that Erik/Elsa and I are working toward mutually agreed upon goals.		4.93 (1.67)		54.0
I felt that Erik/Elsa and I agree on what is important for me to work on.		5.13 (1.68)		76.5^l^
I believe the way Erik/Elsa and I are working with my problem is correct.		5.53 (1.36)		75.5^m^
Overall		5.59 (1.23)		102.0^m^

a SCD: subjective cognitive decline.

b MCI: mild cognitive impairment.

c PEU: perceived ease of use.

d PU: perceived usefulness.

e PEN: perceived enjoyment.

f PC: perceived control.

g ITI: intention to interact.

h PSA: perceived session alliance.

i 7-point Likert scales were used from 1=strongly disagree to 7=strongly agree.

j V is the test statistic of the Wilcoxon signed rank with test value 4.

k *P*<.001.

l *P*<.05.

m *P*<.01.

Results indicate that all mean values lie above the neutral scale value of 4. Moreover, Wilcoxon signed rank tests indicate that the median of perceived ease of use and two items of the session alliance inventory (items 1 and 2) were significantly higher compared to the neutral test value of 4. Additionally, the median values of perceived usefulness as a reminder (PU1), perceived enjoyment, perceived control, and the overall session alliance inventory items were also rated highly. The remaining construct items (perceived usefulness as a motivator [PU2] and intention to interact) still had medians above the value of 4. Therefore, it can be concluded that participants evaluated the CAs Elsa/Erik favorably.

### Qualitative Interview Feedback

**Figure 4.**
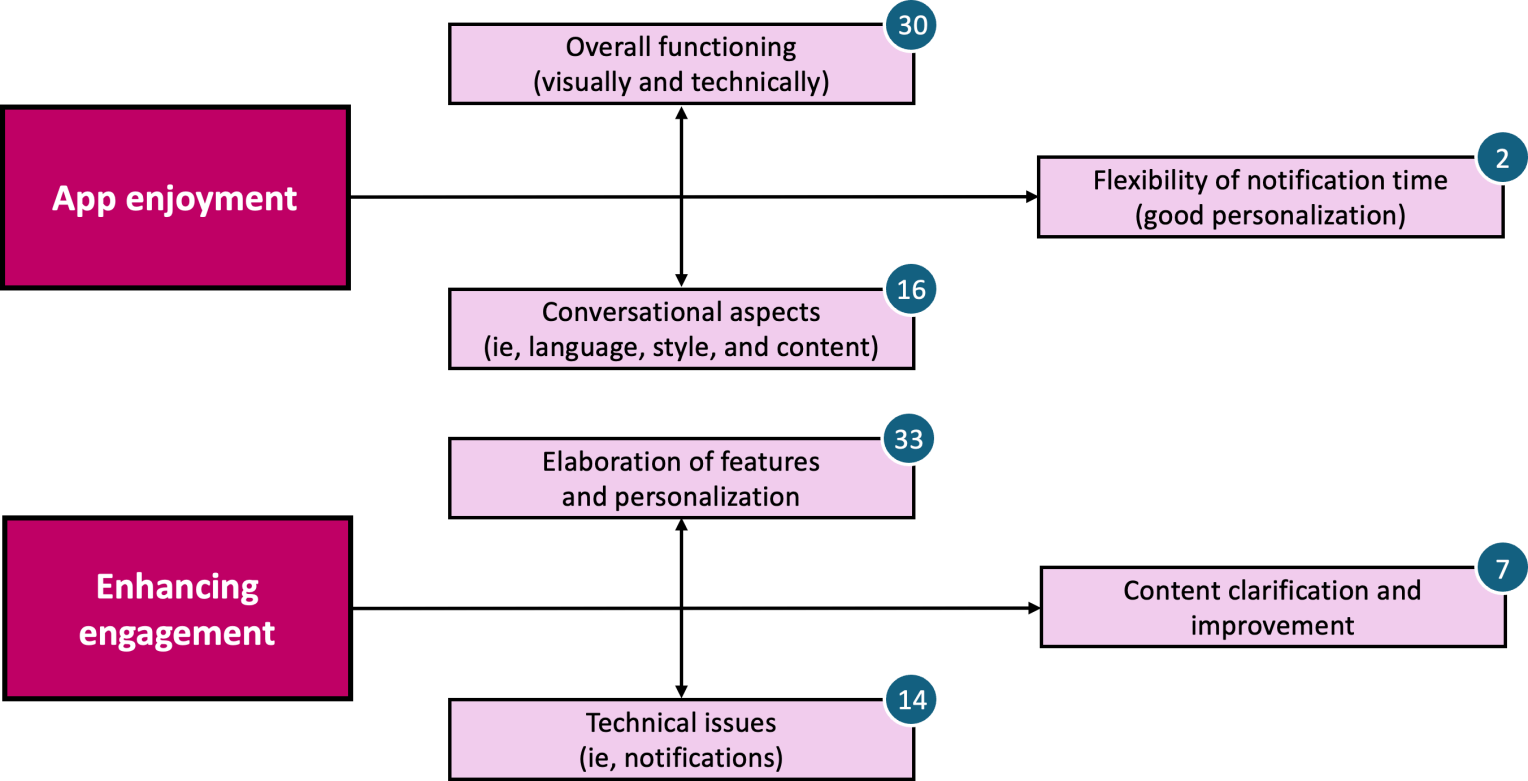
Qualitative results of the 2-week proof-of-concept study conducted with a smartphone-based CA among older adults with SCD or MCI. This presents a thematic map of the main emerging themes, app enjoyment and enhancing engagement, from the qualitative interviews. Note: Numbers in circles represent the frequency count, or how many times the participant theme and its concept were mentioned by participants. Some participants mentioned multiple examples of a single theme during their interview, which then led to multiple frequency counts being identified during thematic analysis. CA: conversational agent; MCI: mild cognitive impairment; SCD: subjective cognitive decline.

To complement the quantitative findings, all participants were contacted postintervention for a short semistructured interview (approximately 10 min). Results focusing on (1) positive feedback, (2) potential for improvement, and (3) open remarks (cf questions mentioned in the Methods) were thematically analyzed and coded. Figure 4, therefore, presents a thematic map of two overarching themes—app enjoyment and enhancing engagement—along with related subthemes and their frequency counts. Multimedia Appendix 3further provides direct participant quotes to illustrate each subtheme and clarify sentiment.

Participants generally appreciated the overall functioning of the app (both visually and technically), with several describing it as “easy to use” (P06, P07, P12, and P17), “appealing, nothing to criticize” (P14), or “the design was ok” (P10, P11, and P12). Participants also responded positively to the tone and conversational style of the agent: “There were no accusations if something wasn’t done, which was very pleasant” (P07), but also highlighted the need for adaptations for older adults: “The language was quite youthful, it should be adjusted since the app is for older people” (P09). The timing and delivery of notifications also emerged as an area for improvement, with several users noting they had missed prompts or found the timing suboptimal: “It might be better if the notifications came in the morning […] especially if you are still working” (P03, P04, and P05).

## Discussion

### Principal Results

#### Overview

This proof-of-concept study investigated the plausibility and acceptability of a smartphone-based JITAI delivered via a rule-based CA (Elsa/Erik) among older adults with SCD or MCI. While our intervention draws on the well-established tradition of relational agents [20,66], it introduces several novel elements: integrating (1) a JITAI logic delivered via a relational agent; (2) embedding a multicomponent, holistic set of lifestyle interventions; and (3) tailoring the JITAI and relational agent to minimize burden for a vulnerable population such as cognitively impaired older adults.

#### Delivering JITAI Logic via a CA

Overall, participants showed high engagement with the JITAI decision-making flow that included daily conversational check-ins to assess states of vulnerability, receptivity, and adherence. Across the 14-day intervention, the overall response rate to the initial question assessing the state of vulnerability amounted to 81%, suggesting strong adherence to the CA-initiated conversational turns (RQ1). Vulnerability was then reported in 27% (n=4.07) of interactions, and 100% of vulnerable participants responded to the follow-up receptivity notification. Notably, 69% (n=2.86) of those classified as receptive also reported completing a suggested activity. Moreover, participants generally reported a positive experience interacting with the CA, as reflected in their ratings of the working alliance. Taken together, these findings support the plausibility of delivering a JITAI via a CA.

#### Toward Personalized Multicomponent, Holistic Lifestyle Intervention

The JITAI intervention delivered lifestyle recommendations across 7 distinct domains of modifiable risk factors relevant to dementia prevention and healthy aging: exercise, balanced diet, sleep, cognitive exercises, movement, social engagement, and hydration [9]. Analysis across domains revealed considerable variation in momentary vulnerability: for example, cognitive exercise emerged as the area with the highest vulnerability, whereas sleep had the lowest reported vulnerability, with only one individual indicating a need for behavioral support on relevant days. On the one hand, this supports the value of a multidomain approach, as it allows tailoring a JITAI to varying individual needs. On the other hand, it suggests that future versions of the JITAI should be more flexible to allow users to select focal domains or automatically personalize content based on historical preferences. Indeed, participants also mentioned that providing supporting information regarding suggested activities or alternatives for certain activities in case they could not complete a specific task on a given day, but still wished to engage, would have been appreciated.

#### Tailoring the Intervention to Prioritize Minimal Burden for Vulnerable Intervention

Results show that participants generally responded positively to the rule-based CA-delivered JITAI. Quantitative and qualitative feedback confirmed perceived ease of use, perceived usefulness (ie, reminders of the health-promoting behavior), enjoyment, and an overall positive working alliance (ie, participant-CA relationship). Taken together, these findings support the usability and acceptability of a structured, CA-delivered JITAI designed for low-burden interactions (RQ2).

However, the findings of this study also highlight several critical aspects related to the deployment of JITAIs in older adults with varying degrees of cognitive impairment, such as MCI and most likely mild Alzheimer disease. One of the most significant considerations is the adequacy of the hardware used to deliver the intervention. As observed, two participants were excluded due to outdated hardware or technical issues, underscoring the importance of future studies to ensure that the technology used is sufficiently robust and up to date [67]. The interview study also revealed a high frequency of mentions regarding issues with notifications. Overall, this emphasizes the need for addressing potential hardware challenges in future studies, as these issues were not related to participant behavior but to technical malfunctions, which is also a key takeaway from our proof-of-concept study.

### Limitations and Future Research Directions

This proof-of-concept study was deliberately designed as a formative, discovery-oriented investigation rather than a hypothesis-driven trial. This said, no a priori benchmarks for adherence or acceptability were defined, given the novelty of the target population and the limited existing evidence on smartphone-based and CA-driven JITAIs in individuals with SCD or MCI. Instead, this design allowed for open-ended observations of use patterns and participant responses to interventions. While this approach supported initial insights into the plausibility and user engagement of the JITAI, future studies will benefit from the use of literature- or theory-informed benchmarks to support clearer interpretability and “go/no-go” decision-making as suggested, for instance, by emerging frameworks such as the Digital Therapeutics Real-World Evidence framework [68].

Another key limitation of the current JITAI design lies in its reliance on self-reported data only to assess both states of vulnerability, receptivity, and adherence. While the target population—individuals with SCD and MCI—is generally capable of providing momentary self-reports over short timeframes, this approach may not scale to individuals with more advanced cognitive decline [45]. To minimize cognitive burden, we limited the recall window to same-day behaviors and used simple, structured prompts. Nevertheless, future iterations of the intervention could benefit from the integration of passive sensing modalities (eg, step count, sleep patterns, location-based context, or even social interaction cues from Bluetooth proximity) to reduce reliance on self-reports and enable more accurate, inclusive data collection. Wearable devices, such as smartwatches or fitness bands, hold particular promise for older adults with cognitive impairments [69]. In addition to enabling continuous, real-time tracking of health behaviors like physical activity, sleep wearables offer a more reliable notification interface: unlike smartphones, which may be overlooked or out of reach, wearables provide discreet, wrist-based notifications through visual cues or haptic feedback, increasing the likelihood that prompts are noticed and acted upon [70].

Further, this early-stage proof-of-concept study was designed to investigate plausibility-related outcomes such as acceptability, usability, and engagement, rather than to demonstrate broad effectiveness. The outcomes observed here reflect the experiences of a predominantly female and technology-aware sample, reflecting common patterns in research study participation, but limit generalizability across genders.

Additionally, embedding the JITAI into a stepped-care program or study with in-person contacts, as a form of blended treatment [71], would allow for additional collection of proxy (or “other”) reports from caregivers or clinicians, which could serve as alternative data source to strengthen the reliability of the decision logic. A blended approach could also offer the necessary support to enhance the overall effectiveness and user experience with the hardware [72,73].

The use of a rather simple, low-burden rule-based CA further constrained the depth and flexibility of user interactions. Participants could only select from predefined response options, and the CA delivered one daily activity suggestion based solely on binary assessments of vulnerability and receptivity. This approach did not accommodate individual preferences or provide alternative options if the suggested behavior was, for instance, irrelevant (cf the low vulnerability reported for days with a sleep intervention). Indeed, several participants expressed a desire for more contextual flexibility and choice. While this JITAI was intentionally designed to reduce cognitive load and mitigate confusion or misinformation, its limited adaptability also constrained the intervention’s alignment with the defining characteristics of a “true” JITAI, which requires nuanced, context-sensitive tailoring over time. Future research may address this limitation by exploring hybrid architectures that retain the safety and clarity of rule-based logic while incorporating the adaptive potential of generative artificial intelligence (eg, large language models). Such models may allow for richer personalization, deeper therapeutic engagement, and more responsive intervention delivery—provided that appropriate safeguards and oversight mechanisms are in place to preserve safety and therapeutic integrity for cognitively vulnerable users [74,75].

### Conclusions

The multicomponent approach used in this study, which integrates various aspects of health such as sleep, hydration, physical fitness, and cognitive exercises, is particularly promising for enhancing the QoL in older adults. By addressing multiple dimensions of well-being simultaneously, this approach has the potential to produce more comprehensive and sustained benefits compared to interventions that focus on a single health aspect. For older adults, maintaining a balance across these domains is crucial, as deficits in one area, like poor sleep or inadequate hydration, can negatively impact other aspects of health, such as cognitive function or physical mobility. By promoting holistic health behaviors, the JITAI not only supports cognitive health but also contributes to overall well-being, potentially delaying the onset of more severe impairments and enhancing daily functioning. This integrated strategy aligns with current geriatric care principles, which emphasize the importance of a well-rounded approach to aging and hold great promise for improving the QoL in this population.

## Supplementary material

10.2196/66885 Multimedia Appendix 1 Preintervention questionnaire (German).

10.2196/66885 Multimedia Appendix 2 Postintervention questionnaire (German).

10.2196/66885 Multimedia Appendix 3 Code groups and participant quotes (translated in English).

10.2196/66885 Checklist 1 CONSORT (Consolidated Standards of Reporting Trials) checklist (English).

## Abbreviations

CA: conversational agent
CONSORT: Consolidated Standards of Reporting Trials
JITAI: just-in-time adaptive intervention
MCI: mild cognitive impairment
MoCA: Montreal Cognitive Assessment
QoL: quality of life
RQ: research question
SCD: subjective cognitive decline
