# Supplementary material for: Toward a Smartphone-Based and Conversational Agent–Delivered Just-in-Time Adaptive Holistic Lifestyle Intervention for Older Adults Affected by Cognitive Decline: Two-Week Proof-of-Concept Study
Source: JMIR Form Res. 2025 Jul 28;9:e66885. doi: 10.2196/66885 (PMC12303554; doi:10.2196/66885)
Supplement: Multimedia Appendix 1 [file formative-v9-e66885-s001.docx]

**Fragebogen**

*JIATI-MCI in der interdisziplinären Memory Clinic Bern*

| Teilnehmer-ID |  | Datum |  | VL |  |
| --- | --- | --- | --- | --- | --- |

| **1** | **Ich besitze …** |
| --- | --- |
|  | ☐ Apple iPhone ☐ Android Smartphone  ☐ Sonstiges : ___________________________________________________ |

| **2** | **Ich nutze mein Smartphone …** | | | | |
| --- | --- | --- | --- | --- | --- |
|  | ☐ niemals | ☐ ca. 1 x  pro Woche | ☐ mehrmals  pro Woche | ☐ ca. 1 x  pro Tag | ☐ mehrmals  am Tag |

| **3** | **Welche Aussage trifft am Ehesten auf Sie auf?** |
| --- | --- |
|  | ☐ Ich trage mein Handy **die ganze Zeit** nah bei mir, auch zuhause.  ☐ Ich trage mein Handy nur dann bei mir, wenn ich **unterwegs** bin (z.B. im Garten, beim Einkaufen, etc. …).  ☐ Ich nehme mein Handy nur dann zur Hand, wenn ich es **brauche** (z.B. zum telefonieren). |

| **4** | **Ich nutze mein Smartphone um …** | | | | | | | | |  | |  | |  |  |
| --- | --- | --- | --- | --- | --- | --- | --- | --- | --- | --- | --- | --- | --- | --- | --- |
|  |  | | | **nie** | | **Ca. 1x/Woche** | | **Mehrmals pro Woche** | | **Ca. 1x/Tag** | | **Mehrmals am Tag** | | **Ich weiss nicht** | |
| … zu telefonieren | | | | ☐ | | ☐ | | ☐ | | ☐ | | ☐ | | ☐ | |
| … Fotos aufzunehmen | | | | ☐ | | ☐ | | ☐ | | ☐ | | ☐ | | ☐ | |
| … Spiele zu spielen | | | | ☐ | | ☐ | | ☐ | | ☐ | | ☐ | | ☐ | |
| … Informationen im Internet zu recherchieren | | | | ☐ | | ☐ | | ☐ | | ☐ | | ☐ | | ☐ | |
| … mit Freunden und Bekannten zu «chatten» (d.h. Nachrichten hin- und her zu schicken) | | | | ☐ | | ☐ | | ☐ | | ☐ | | ☐ | | ☐ | |
|  | | | |  | |  | |  | |  | |  | |  | |
|  | |  |  | |  | |  | |  | |  | |  | | |

| **4** | **Ich nutze mein Smartphone um …** | | | |  |  |  |  |
| --- | --- | --- | --- | --- | --- | --- | --- | --- |
|  |  | **nie** | **Ca. 1x/Woche** | **Mehrmals pro Woche** | **Ca. 1x/Tag** | **Mehrmals am Tag** | **Ich weiss nicht** | |
| … Musik zu hören | | ☐ | ☐ | ☐ | ☐ | ☐ | ☐ | |
| … Gesundheitsdaten (z.B. Anzahl Schritte) aufzuzeichnen | | ☐ | ☐ | ☐ | ☐ | ☐ | ☐ | |
| … die Wettervorhersage anzuschauen | | ☐ | ☐ | ☐ | ☐ | ☐ | ☐ | |
| … E-Mails zu lesen | | ☐ | ☐ | ☐ | ☐ | ☐ | ☐ | |
| … E-Mails zu beantworten | | ☐ | ☐ | ☐ | ☐ | ☐ | ☐ | |
| … als Routenplanen / um Adressen und Orte zu finden | | ☐ | ☐ | ☐ | ☐ | ☐ | ☐ | |
| … meine Agenda / Kalender einzusehen | | ☐ | ☐ | ☐ | ☐ | ☐ | ☐ | |
