# Supplementary material for: Toward a Smartphone-Based and Conversational Agent–Delivered Just-in-Time Adaptive Holistic Lifestyle Intervention for Older Adults Affected by Cognitive Decline: Two-Week Proof-of-Concept Study
Source: JMIR Form Res. 2025 Jul 28;9:e66885. doi: 10.2196/66885 (PMC12303554; doi:10.2196/66885)
Supplement: Multimedia Appendix 2 [file formative-v9-e66885-s002.docx]

**Fragebogen: Post**

*JIATI-MCI in der interdisziplinären Memory Clinic Bern*

| Teilnehmer-ID |  | Datum |  | VL |  |
| --- | --- | --- | --- | --- | --- |

| **1** | **Wie haben Sie die Interaktion mit Elsa/Erik wahrgenommen?** | stimme überhaupt nicht zu | | weder noch | | | stimme  voll und ganz zu | |
| --- | --- | --- | --- | --- | --- | --- | --- | --- |
| **A** | Ich fand die Interaktion Elsa/Erik einfach. | ☐  1 | ☐ 2 | ☐ 3 | ☐ 4 | ☐ 5 | ☐ 6 | ☐ 7 |
| **B** | Die Interaktion mit Elsa/Erik hat mir Spass gemacht. | ☐  1 | ☐ 2 | ☐ 3 | ☐ 4 | ☐ 5 | ☐ 6 | ☐ 7 |
| **C** | Ich fand es nützlich von Elsa/Erik an die Aufgaben/Aktivitäten erinnert zu werden. | ☐  1 | ☐ 2 | ☐ 3 | ☐ 4 | ☐ 5 | ☐ 6 | ☐ 7 |
| **D** | Elsa/Erik hat mich motiviert die Aufgaben/Aktivitäten durchzuführen. | ☐  1 | ☐ 2 | ☐ 3 | ☐ 4 | ☐ 5 | ☐ 6 | ☐ 7 |
| **E** | Ich konnte den Dialog mit Elsa/Erik stets kontrollieren. | ☐  1 | ☐ 2 | ☐ 3 | ☐ 4 | ☐ 5 | ☐ 6 | ☐ 7 |
| **F** | Ich würde gerne weiterhin mit Elsa/Erik interagieren. | ☐  1 | ☐ 2 | ☐ 3 | ☐ 4 | ☐ 5 | ☐ 6 | ☐ 7 |

| **2** | **Bitte beurteilen Sie Ihre Beziehung mit Elsa/Erik?** | Nie | |  | | | Immer | |
| --- | --- | --- | --- | --- | --- | --- | --- | --- |
| **A** | Elsa/Erik und ich haben uns respektiert. | ☐  1 | ☐ 2 | ☐ 3 | ☐ 4 | ☐ 5 | ☐ 6 | ☐ 7 |
| **B** | Ich hatte das Gefühl, dass Elsa/Erik mich wertschätzt. | ☐  1 | ☐ 2 | ☐ 3 | ☐ 4 | ☐ 5 | ☐ 6 | ☐ 7 |
| **C** | Ich hatte das Gefühl, dass Elsa/Erik sich um mich kümmert, auch wenn ich Dinge tue, die Elsa/Erik nicht gut findet. | ☐  1 | ☐ 2 | ☐ 3 | ☐ 4 | ☐ 5 | ☐ 6 | ☐ 7 |
| **D** | Elsa/Erik und ich arbeiteten auf gemeinsam vereinbarte Ziele hin. | ☐  1 | ☐ 2 | ☐ 3 | ☐ 4 | ☐ 5 | ☐ 6 | ☐ 7 |
| **E** | Elsa/Erik und ich sind uns einig, woran es für mich wichtig ist zu arbeiten. | ☐  1 | ☐ 2 | ☐ 3 | ☐ 4 | ☐ 5 | ☐ 6 | ☐ 7 |
| **F** | Ich denke, dass unsere Herangehensweise mein Problem zu lösen korrekt ist. | ☐  1 | ☐ 2 | ☐ 3 | ☐ 4 | ☐ 5 | ☐ 6 | ☐ 7 |

| **3** | **Was fanden Sie besonders gut an der Interaktion mit Elsa/Erik?** | | | | | |  | |
| --- | --- | --- | --- | --- | --- | --- | --- | --- |
|  |  |  |  |  |  |  |  |  |
|  |  |  |  |  |  |  |  |  |
|  |  |  |  |  |  |  |  |  |

| **4** | **Was müsste unbedingt an der Interaktion mit Elsa/Erik verbessert werden?** | | | | | |  | |
| --- | --- | --- | --- | --- | --- | --- | --- | --- |
|  |  |  |  |  |  |  |  |  |
|  |  |  |  |  |  |  |  |  |
|  |  |  |  |  |  |  |  |  |

| **5** | **Haben Sie weitere Kommentare bzw. Verbesserungsvorschläge zum Studienablauf?** | | | | | |  | |
| --- | --- | --- | --- | --- | --- | --- | --- | --- |
|  |  |  |  |  |  |  |  |  |
|  |  |  |  |  |  |  |  |  |
|  |  |  |  |  |  |  |  |  |

| **6** | **Offenes Feedback und Details zum Smartphone und mobilien Betriebssystem.** | | | | | | | |
| --- | --- | --- | --- | --- | --- | --- | --- | --- |
|  |  |  |  |  |  |  |  |  |
|  |  |  |  |  |  |  |  |  |
|  |  |  |  |  |  |  |  |  |

Vielen Dank für Ihre Teilnahme an unserer Studie!

Ihr Studienteam
