# Supplementary material for: Toward a Smartphone-Based and Conversational Agent–Delivered Just-in-Time Adaptive Holistic Lifestyle Intervention for Older Adults Affected by Cognitive Decline: Two-Week Proof-of-Concept Study
Source: JMIR Form Res. 2025 Jul 28;9:e66885. doi: 10.2196/66885 (PMC12303554; doi:10.2196/66885)
Supplement: Multimedia Appendix 3 [file formative-v9-e66885-s003.pdf]

Code		Selected participant quotes
Content	Content was confusing	*P17: "The 'Thank you' when saying goodbye was a little confusing".*
	Helpful	*P04: "The reminder aspect was very helpful."*<br>*P06: "The core idea of the app is very good."*<br>*P13: "The app always suggested good things, like drinking enough water."*
	More features	*P02: "Perhaps an option where you can write something yourself would be better, especially for older people who are looking for contact."*<br>*P09: "It shouldn't be like a strict schedule, if you don't have time for sports on Monday, you should be able to do it another day — perhaps add a 'postpone' button."*<br>*P11: "It could be expanded by adding links to studies, for example, explaining why exercise or similar is good, especially for those interested."*<br>*P11: "If it lasted longer than just two weeks, personal conversations would certainly be helpful."*
	More motivating	*P02: "The app only motivated me to do something a little, only once."*<br>*P04: "The suggestions weren't new, just things that are already paid attention to anyway."*<br>*P15: "Not all suggestions are equally helpful."*
	Style of response	*P02: "The responses were only positive, there should also be negative responses."*<br>*P07: "There were no accusations if something wasn't done, which was very pleasant."*<br>*P09: "The language was quite youthful, it should be adjusted since the app is for older people."*<br>*P15: "The questions were very sympathetic."*
Technical	Technical issues	*P09: "When opening the app, only the picture of Elsa always came up, which was a bit confusing since Erik was chosen as the conversation partner. Perhaps include both pictures there."*<br>*P09: "On July 24th, the response option was contradictory. I indicated that something wasn't done, but the app said 'well done'."*<br>*P17: "The font could be adjusted, maybe made larger."*
	Positive visuals	*P10, 11 & 12: "The design was okay."*<br>*P14: "The design was appealing, nothing to criticize."*
	Worked well	*P09: "The operation was good."*<br>*P13: "Notifications were received, everything always worked".*
	No notifications	*P06: "Also, did not receive any notifications, so did nothing for the first three days."*<br>*P14: "Didn't receive notifications, I always went into the app myself in the afternoon."*
Usability	Different timing options	*P03, 04 & 05: "It might be better if the notifications came in the morning, as you would have more time to plan your day (especially if you are still working)."*<br>*P11: "The time in which you have to respond is sometimes predetermined."*
	Liked timing flexibility	*P02: "The ability to choose between two times provided flexibility in implementing the suggestions."*<br>*P17: "The times are very good, I don't always have lunch at 12:00 but sometimes later."*
	Easy to use	*P06, 07, 12 & 17: "The app was very easy to use."*<br>*P13: "Operation was easy."*

**Perspective (app)**	Good for others	*P04: "Apps are good for older people to stay connected and not miss out, but I personally don't find apps that great."*<br>*P14: "For a person who is still doing well, the app doesn't make much sense. For people who need more reminders, it's a good thing."*<br>*P15: "It's definitely doable and probably helpful for people with limitations."*
	More elaborate	*P02: "The design is simple; it could be made a bit different, more elaborate or something like that."*<br>*P11: "Operation was also very simple."*<br>*P12: "The app was nothing special. Not sure if it really makes a difference."*
	Good app	*P04: "The freedom the app provided was good. It didn't dictate anything about carrying out the interventions."*<br>*P07: "Found the app to be a very good thing. It really does you good."*<br>*P11: "A very lenient app, with a very friendly tone. The pressure the app applies is also appropriate."*
	Bad app	*P05: "Did not find the app good. I also didn't receive notifications, so sometimes I couldn't respond at all. The app too simple and very limiting."*<br>*P12: "Didn't take much interest in the app and didn't think much about it."*
